# Quantitative susceptibility mapping reveals alterations of dentate nuclei in common types of degenerative cerebellar ataxias

**DOI:** 10.1093/braincomms/fcab306

**Published:** 2022-01-13

**Authors:** Andreas Deistung, Dominik Jäschke, Rossitza Draganova, Viktor Pfaffenrot, Thomas Hulst, Katharina M. Steiner, Andreas Thieme, Ilaria A. Giordano, Thomas Klockgether, Sinem Tunc, Alexander Münchau, Martina Minnerop, Sophia L. Göricke, Jürgen R. Reichenbach, Dagmar Timmann

**Affiliations:** 1 University Clinic and Outpatient Clinic for Radiology, Department for Radiation Medicine, University Hospital Halle (Saale), Halle (Saale), Germany; 2 Medical Physics Group, Institute of Diagnostic and Interventional Radiology, Jena University Hospital, Jena, Germany; 3 Department of Neurology and Center for Translational Neuro- and Behavioral Sciences (C-TNBS), Essen University Hospital, Essen, Germany; 4 Erwin L. Hahn Institute for Magnetic Resonance Imaging, University Duisburg-Essen, Essen, Germany; 5 Erasmus University College, Erasmus School of Social and Behavioural Sciences, Erasmus University Rotterdam, Rotterdam, The Netherlands; 6 Department of Neurology, University Hospital Bonn, Bonn, Germany; 7 German Center for Neurodegenerative Diseases (DZNE), Bonn, Germany; 8 Institute of Systems Motor Science, University of Lübeck, Lübeck, Germany; 9 Department of Neurology, University of Lübeck, Lübeck, Germany; 10 Institute of Neuroscience and Medicine (INM-1), Research Center Juelich, Juelich, Germany; 11 Department of Neurology, Center for Movement Disorders and Neuromodulation, Medical Faculty, Heinrich-Heine University, Düsseldorf, Germany; 12 Institute of Clinical Neuroscience and Medical Psychology, Medical Faculty, Heinrich-Heine University Düsseldorf, 40225 Duesseldorf, Germany; 13 Institute of Diagnostic and Interventional Radiology and Neuroradiology, Essen University Hospital, Essen, Germany

**Keywords:** ataxia, cerebellum, MRI, quantitative susceptibility mapping, iron

## Abstract

The cerebellar nuclei are a brain region with high iron content. Surprisingly, little is known about iron content in the cerebellar nuclei and its possible contribution to pathology in cerebellar ataxias, with the only exception of Friedreich’s ataxia. In the present exploratory cross-sectional study, quantitative susceptibility mapping was used to investigate volume, iron concentration and total iron content of the dentate nuclei in common types of hereditary and non-hereditary degenerative ataxias. Seventy-nine patients with spinocerebellar ataxias of types 1, 2, 3 and 6; 15 patients with Friedreich’s ataxia; 18 patients with multiple system atrophy, cerebellar type and 111 healthy controls were also included. All underwent 3 T MRI and clinical assessments. For each specific ataxia subtype, voxel-based and volumes-of-interest-based group analyses were performed in comparison with a corresponding age- and sex-matched control group, both for volume, magnetic susceptiblity (indicating iron concentration) and susceptibility mass (indicating total iron content) of the dentate nuclei. Spinocerebellar ataxia of type 1 and multiple system atrophy, cerebellar type patients showed higher susceptibilities in large parts of the dentate nucleus but unaltered susceptibility masses compared with controls. Friedreich’s ataxia patients and, only on a trend level, spinocerebellar ataxia of type 2 patients showed higher susceptibilities in more circumscribed parts of the dentate. In contrast, spinocerebellar ataxia of type 6 patients revealed lower susceptibilities and susceptibility masses compared with controls throughout the dentate nucleus. Spinocerebellar ataxia of type 3 patients showed no significant changes in susceptibility and susceptibility mass. Lower volume of the dentate nuclei was found to varying degrees in all ataxia types. It was most pronounced in spinocerebellar ataxia of type 6 patients and least prominent in spinocerebellar ataxia of type 3 patients. The findings show that alterations in susceptibility revealed by quantitative susceptibility mapping are common in the dentate nuclei in different types of cerebellar ataxias. The most striking changes in susceptibility were found in spinocerebellar ataxia of type 1, multiple system atrophy, cerebellar type and spinocerebellar ataxia of type 6. Because iron content is known to be high in glial cells but not in neurons of the cerebellar nuclei, the higher susceptibility in spinocerebellar ataxia of type 1 and multiple system atrophy, cerebellar type may be explained by a reduction of neurons (increase in iron concentration) and/or an increase in iron-rich glial cells, e.g. microgliosis. Hypomyelination also leads to higher susceptibility and could also contribute. The lower susceptibility in SCA6 suggests a loss of iron-rich glial cells. Quantitative susceptibility maps warrant future studies of iron content and iron-rich cells in ataxias to gain a more comprehensive understanding of the pathogenesis of these diseases.

## Teaser

Deistung *et al*. report that dentate nuclei exhibited prominent increases in iron concentration in spinocerebellar ataxia type 1 and multiple system atrophy, cerebellar type and decreases in iron concentration and volume in spinocerebellar ataxia type 6, whereas spinocerebellar ataxia types 2 and 3 and Friedreich’s ataxia showed less distinct alterations.

## Graphical Abstract

**Graphical Abstract:**
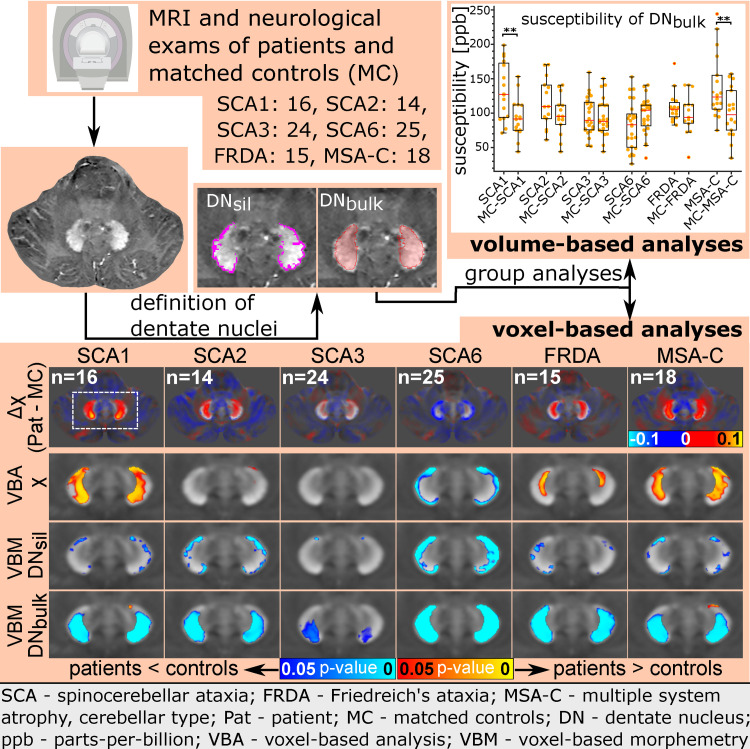

See Harding and Ward (https://doi.org/10.1093/braincomms/fcac007) for a scientific commentary on this article.

## Introduction

Abnormal iron accumulation in the brain plays an important role in many neurodegenerative disorders.^1^ Although detailed knowledge of the molecular and cellular mechanisms of brain iron accumulation and iron-related neurodegeneration is limited, iron-induced oxidative stress is one likely cause of neuronal cell death.^1^ Iron accumulation in the brain is not limited to rare hereditary disorders, which are grouped under the term neurodegeneration with brain iron accumulation (NBIA).^2^ It is also observed in more common neurodegenerative diseases such as Huntington disease, Parkinson disease and Alzheimer disease, as well as multiple sclerosis.^1^ In most of these diseases, iron accumulates in the basal ganglia.^1,3^ Here, iron content is already physiologically high and is well known to increase with age.^1,4,5^

Iron accumulation in the brain is also a hallmark of multiple system atrophy (MSA).^6^ In MSA, iron accumulation is not limited to the basal ganglia but also includes the dentate nuclei,^6,7^ another brain region with physiologically high and age-dependent iron content located in the cerebellum.^5^ Surprisingly, little is known about iron metabolism and its potential contribution to pathology in hereditary cerebellar ataxias. The only exception is Friedreich’s ataxia (FRDA), in which reduction of frataxin leads to changes in cellular iron homeostasis.^8^ However, even in NBIA, iron accumulation is rarely associated with genes directly involved in iron metabolism.^2^ More commonly, iron accumulation occurs indirectly, especially in connection with microgliosis and inflammation.^9^ Pronounced microgliosis has been described in spinocerebellar ataxia type 1 (SCA1).^10^ Thus, iron accumulation may also play a role in the pathogenesis of hereditary ataxias other than FRDA. Improved knowledge of potential iron accumulation in ataxias may be of clinical value as brain iron chelation therapy has become available,^11^ and—perhaps more importantly—in identifying potential biomarkers.

MRI provides a unique opportunity to study brain iron concentration *in vivo*. Susceptibility weighted imaging (SWI), a qualitative MRI technique sensitive to iron deposition, has been used in the past to visualize the cerebellar nuclei.^12^ SWI, however, is subject to several limitations, including its non-quantitative nature and the inherent blooming effect of iron deposits on the images. These limitations are largely overcome by its offspring, the so-called quantitative susceptibility mapping (QSM),^13^ which additionally allows quantification of iron concentration *in vivo*.^14^

In the present exploratory cross-sectional study, QSM was used to determine iron concentration and total iron content in the largest of the cerebellar nuclei, the dentate nucleus, in different forms of ataxia. Findings in FRDA were compared with findings in the most common forms of dominantly inherited ataxias [spinocerebellar ataxia types 1, 2, 3 and 6 (SCA1,2,3,6)] and a common form of non-hereditary degenerative ataxia [multiple system atrophy, cerebellar type (MSA-C)]. QSM revealed different patterns of abnormalities in the dentate nuclei. Abnormalities in susceptibility were most pronounced in SCA1, MSA-C and SCA6 patients. While susceptibility was significantly higher in SCA1 and MSA-C, it was significantly lower in SCA6 compared with healthy controls. Smaller changes in susceptibility were found in the dentate nuclei in FRDA and SCA2 and no change in SCA3 patients. Our data suggest that changes in iron concentration may contribute to the pathogenesis of a subset of cerebellar ataxias or at least be a result or indicator of the underlying pathology.

## Materials and methods

### Study participants

Eighty-four patients with spinocerebellar ataxias (SCA1, SCA2, SCA3, SCA6), 15 patients with FRDA and 19 patients with MSA-C as well as 126 healthy controls underwent 3 T MRI and clinical assessments at the University Hospital Essen in the period of March 2016–October 2018. Six patients and fifteen healthy subjects were excluded due to incidental pathological findings, incomplete MRI data due to measurement interruptions or unacceptable artefacts due to motion. A total of 16 SCA1, 14 SCA2, 24 SCA3, 25 SCA6, 15 FRDA and 18 MSA-C patients were included. The study was approved by the internal Ethics Committee of the Essen University Hospital and was conducted in accordance with the Declaration of Helsinki. Written informed consent was obtained from all participating subjects.

Clinical history was obtained from both patients and controls. Genetic diagnoses, including repeat lengths of the affected allele, were confirmed in all patients with hereditary ataxia. MSA-C patients met the criteria of probable or possible MSA-C.^15^ Healthy controls had no current or past history of neurological or psychiatric disorders. They had no family history of hereditary disease but were not genetically screened. Clinical scores were obtained for patients and controls based on the Scale for the Assessment and Rating of Ataxia (SARA, range: 0–40),^16^ the Inventory of Non-Ataxia Signs (INAS, range: 0–16)^17^ and the SpinoCerebellar Ataxia Functional Index (SCAFI).^18^ Higher SARA and INAS scores and lower SCAFI scores indicate worse performance.

For each ataxia subgroup, an age- and sex-matched control subgroup was selected from the entire sample of healthy controls. Unpaired *t*-tests were applied to probe statistically significant differences in age, SARA and SCAFI between patients and control groups.

### MRI data acquisition

MRI data were collected with a human whole-body combined MRI–PET system (Siemens Healthineers, Erlangen, Germany), operating at a magnetic field strength of 3 T, by using a 16-channel head array coil (Siemens Healthineers). Multi-echo, 3D gradient-echo (GRE) imaging {four echoes, monopolar readout, echo-times (TEs)_1–4 _= 6.47 ms/17.23 ms/27.99 ms/38.75 ms, repetition time (TR) = 62 ms, flip angle (FA) = 17°, bandwidth (BW)_1–4_ = 120 Hz/px, phase encoding direction: right/left, acquisition matrix = 384 × 324 × 160, voxel size = 0.5 mm × 0.5 mm × 0.5 mm, parallel imaging (GRAPPA) undersampling along the phase encoding direction [factor (*R*) = 2, reference lines = 48], 75% partial Fourier along slice encoding direction, acquisition time (TA) = 13:09 min:s} was carried out in transverse-to-coronal orientation for subsequent quantitative susceptibility mapping. Saturation pulses were positioned inferior and superior to the field-of-view (FoV) to avoid non-local artefacts due to pulsatile blood flow in vessels close to the cerebellum. In addition, whole-head T_1_-weighted (T1w) MRI data sets were collected with a magnetization-prepared rapid gradient-echo (MP-RAGE) sequence [isotropic voxel size of 1 mm, TE = 3.26 ms, TR = 2530 ms, inversion time (TI) = 1100 ms, FA = 7°, acquisition matrix = 256 × 256 × 176, BW = 200 Hz/Px, GRAPPA with *R* = 2 and 48 reference lines, TA = 6:24 min:s] for cerebellum-based spatial normalization. Finally, fluid-attenuated inversion recovery (FLAIR) images covering the whole brain were acquired using a 2D sequence (TI = 2500 ms, TE = 94 ms, TR = 9000 ms, FA = 150°, acquisition matrix = 256 × 208, 55 contiguous slices, voxel size = 0.45 mm × 0.45 mm × 3 mm, TA = 3:38 min:s). MP-RAGE and FLAIR images were inspected by a neuroradiologist (S.L.G.). Healthy controls were excluded from the study in cases where brain abnormalities were identified.

### MRI data processing

#### Quantitative susceptibility mapping

A spatially adaptive non-local means denoising algorithm^19^ was applied to the real and imaginary parts of the complex-valued images to mitigate noise. Quantitative susceptibility maps were computed based on these denoised phase images. To this end, the phase images for each echo were unwrapped using a 3D best-path algorithm,^20^ divided by 2*π* · TE_*i*_ to obtain the Larmor frequency variation in Hz and then combined across the different TEs. Background frequency contributions were removed using sophisticated harmonic artefact removal for phase data (SHARP)^21^ with 10 different spherical kernels with varying radii ranging from 1 to 10 voxels and employing a high-pass filter of 0.01 for regularization. Susceptibility mapping was performed based on the SHARP-processed frequency images using homogeneity-enabled incremental dipole inversion (HEIDI).^22^ We referenced all susceptibility maps to the average susceptibility of the brain tissue within the FoV and stated susceptibility values in parts-per-billion (ppb).

#### Segmentation and volume estimation

Two different volumes-of-interest (VOIs) were created. The first VOI was manually traced and followed the silhouette of the dentate nucleus (DN_sil_) as accurately as possible (Supplementary Fig. 1C). DN_sil_ was used as a proxy for the volume of the dentate nucleus with its characteristic corrugated thin walls.^12,23^ Higher susceptibility values of the dentate nuclei on MR susceptibility maps, however, extend beyond these thin walls to include white matter (WM) within the sac formed by the dentate nucleus (see Supplementary Fig. 1A and B)^24,25^ Therefore, the second VOI represents the bulk of the iron-rich region of the dentate nucleus (DN_bulk_) seen on the susceptibility maps (Supplementary Fig. 1D).

The dentate nuclei (DN_sil_) were demarcated on the susceptibility maps by an experienced technician who was blinded to diagnosis and age. The dentate nuclei were manually traced in both cerebellar hemispheres on the axial, sagittal and coronal susceptibility maps using MRICroN (http://people.cas.sc.edu/rorden/mricron/). Drawings were made directly on the susceptibility maps, also incorporating information from the magnitude and SHARP-processed frequency images.

DN_bulk_ was automatically calculated based on the convex hull obtained from DN_sil_. To this end, a Delaunay triangulation was computed separately for each hemisphere using the 3D coordinates of DN_sil_ to create a triangulated mesh based on which the enclosing coordinates of each triangle were determined. The coordinates were then transformed to the original 3D grid and a specific integer value indicating the VOI was assigned at the coordinate positions. The resulting VOI was additionally eroded with a 3 × 3 × 3 box kernel and corrected for possible CSF contributions by excluding voxels in which the corresponding effective transverse relaxation rate was below 15 s^−1^. These VOIs referring to DN_bulk_ were visually inspected and manually corrected as needed.

Volumes and mean susceptibility (*χ*) values were calculated in the two VOIs to assess differences in volume and iron concentration of the dentate nuclei between subgroups of patients and corresponding controls. Dentate nuclei volumes were summed across the left and right hemisphere. Because iron concentration and thus susceptibility could be affected by atrophy, we also examined the susceptibility mass (*χ*_mass_) of DN_sil_ and DN_bulk_ as a measure of total tissue iron content.^26,27^ Similar to Hernandez-Torres *et al*.,^27^ susceptibility mass was calculated by multiplying the non-normalized dentate volume by the mean susceptibility.

For further comparison, the volume of the cerebellum was also determined from the T1w images by using an established automated cerebellar lobule segmentation method.^28^ Based on this segmentation, cerebellar volume was calculated as the sum of all segmented cerebellar lobules, vermis and the cerebellar WM segment.

To control for differences in head size, the total intracranial volume (TIV) was estimated from the T1w images using the standard pre-processing pipeline of the Computational Anatomy Toolbox 12 (CAT12, http://www.neuro.uni-jena.de/cat/).

#### Voxel-based analysis

The SUIT toolbox (v3.2, http://www.diedrichsenlab.org/imaging/suit.htm) was used to pre-process the data for voxel-based analysis (VBA). More specifically, it was used to transfer the individual data sets [susceptibility maps, dentate VOIs (DN_sil_ and DN_bulk_), GM segmentation derived from T1w images] into the SUIT space.^29^ The SUIT toolbox needs a binary mask of the cerebellum to constrain the computation of grey matter (GM) and WM segmentations, as well as to optimally perform registrations and transformations. Although the SUIT processing pipeline is capable to compute such a binary mask, we fed the T1w data into an alternative automatic segmentation approach based on a fully connected convolutional neural network^30^ to compute individual masks of the cerebellum because of superior performance of the neural network approach compared with the SUIT-based cerebellum segmentation approach. The cerebellum segmentations obtained from the neural network were visually inspected and manually corrected as needed. To integrate both the T1w data and the cerebellum mask into the processing pipeline of SUIT, these data were first oriented so that their common origin was located on the anterior commissure–posterior commissure line. Next, segmentation into GM and WM tissue was performed using the T1w data. The segmented GM and WM data were then non-linearly registered to the SUIT template^29^ considering the individual dentate VOI (DN_bulk_) using DARTEL [Statistical Parametric Mapping (SPM), https://www.fil.ion.ucl.ac.uk/spm/].^12^ The GM segmentation and the dentate VOIs (DN_sil_ and DN_bulk_), as well as the susceptibility maps were resliced into the SUIT space (resolution: 0.5 mm isotropic) using the generated flow field and affine transformations. The different voxel resolutions and the possible misalignment between the T1w and GRE data were taken into account by using the transformation obtained by linear registration (6 degrees of freedom) of the GRE magnitude data (average image of the first and second echo) to the T1w data. Since only the header information was adjusted accordingly, only a single reslicing step was applied to the dentate VOIs and the susceptibility map. GM segmenation and dentate VOIs were modulated to compensate for volume changes during the spatial normalization by multiplying the intensity value in each voxel with the Jacobian determinants. Susceptibility maps were resliced into SUIT space without and with modulation by the Jacobian determinants to assess average susceptibility (*χ*) as a proxy for iron concentration and apparent susceptibility mass (${\tilde{\chi }}_{mass}$), a measure accounting for transform-induced volume changes, as a proxy for iron content, respectively. Finally, GM, dentate (DN_sil_, DN_bulk_) and susceptibility data (*χ*, ${\tilde{\chi }}_{mass}$) in the SUIT space were smoothed using a 3D Gaussian kernel of 1 mm full-width at half maximum.

### Statistical analyses

#### Voxel-based susceptibility analysis and voxel-based morphometry

First, the spatially normalized susceptibility maps were averaged over the patient group of a specific ataxia type and over the corresponding control group. For each ataxia type, the average map of the corresponding control group was subtracted from that of the specific patient group to visualize disease-related alterations in magnetic susceptibility in the cerebellar nuclei.

Next, we performed voxel-wise statistical analysis via non-parametric permutation tests (FSL randomise; 5000 permutations) using age as a covariate to identify susceptibility as well as volume differences between the patient groups and their respective control groups. Although the patient and control groups were matched for age, age was included as a covariate because there was a distinct range in age within the groups. Threshold-free cluster enhancement (TFCE),^31^ while controlling for family-wise error rate, revealed significant differences between groups at the *P* < 0.05 level. Given the study objectives, VBA of pre-processed susceptibility maps (see the MRI data processing section) and voxel-based morphometry (VBM) of the dentate nucleus (that is, the analyses of volumetric changes) were limited to a mask of the dentate nuclei provided by the SUIT toolbox, which was manually corrected to ensure that the whole dentate is captured without including the surrounding WM (Supplementary Fig. 2). VBM of the dentate nucleus was performed using DN_sil_ and DN_bulk_ (both were pre-processed as described previously). To account for differences in head size, TIV was additionally included as a covariate in the VBM.

In a similar setting, voxel-wise correlations between magnetic susceptibilities or dentate volumes and SARA score were calculated to identify a possible relationship between dentate nucleus alterations and clinical assessment of disease severity. This was performed in patients only, and age was included as a covariate to account for distinct age variations within each group.

For comparison, VBM of cerebellar GM was also performed to show the distribution of volume loss within the cerebellar cortex in the different types of ataxia. Statistical analysis of cerebellar GM was restricted to cerebellar tissue using the cerebellum mask of the SUIT toolbox. Statistical analysis results for cerebellar GM are visualized on a flat representation of the cerebellum.^32^

#### VOI-based analysis

Brain volumes must be corrected for head size. To this end, residualization was used to account for differences in head size^33,34^ as the volumes of the dentate nuclei are very small and residualization is less affected by systematic and random errors in TIV and dentate volume. Linear regressions between the absolute volumes (DN_sil_, DN_bulk_, cerebellar volume) and the TIVs of the healthy controls yielded the linear functional relationship [v(TIV)]. The residuals of the individual volumes were calculated with respect to their prediction v(TIV) and standardized according to the ones of the whole sample. By using the linear relationship between the VOI and TIV of the control group for the correction, it was assumed that this linear function represents the ‘normal’ relationship between the VOI and TIV, but this relationship is not necessarily maintained in the case of pathology. Consequently, standardized residuals are used as estimates of the volume corrected for head size.

To compare differences in dentate susceptibility as well as dentate and GM volumes (standardized residuals) between the specific patient groups and their corresponding matched control groups, analyses of covariance (ANCOVAs) were applied with disease type (patient versus control) as the group variable and age as a covariate. Age was included as a covariate to account for age variation within subgroups. Partial eta squared, *η^2^*, were calculated to quantify effect sizes.

In a second step, to identify ataxia subgroup-specific differences among dentate susceptibility, dentate volume and cerebellar volume, respectively, an ANCOVA was calculated for each of these parameters with ataxia disease type as group factor and either age or SARA as a covariate. To account for age-related effects, ANCOVA was performed with age as covariate. To account for disease severity, another ANCOVA was calculated with SARA score as a covariate. If the ANCOVA yielded statistical significance, the Sidak test (*α* = 0.05) was applied for *post hoc* analysis.

Finally, VOI measures of volume and dentate susceptibility were correlated with SARA scores for each ataxia subgroup. Again, this was done only in the patient groups, and age was included as a covariate. We considered age because SARA increases with age in SCA1 and SCA6^35^ and dentate susceptibility increases with age in healthy controls.^36–38^

### Data availability

The data supporting the findings of this study are available from the corresponding author upon reasonable request.

## Results

### Demography

Demographic and clinical details are summarized in Table 1. There was no significant difference in age between the patient and control groups, whereas changes in disease severity assessed via SARA and SCAFI were statistically significant.

**Table 1:** Demographic and clinical characteristics of the study population

Patients						
Disease type	SCA1	SCA2	SCA3	SCA6	FRDA	MSA-C
*n*	16	14	24	25	15	18
Age (years)	47.4 (31.6–68.3)	52.1 (33.1–67.1)	53.0 (25.6–74.6)	61.6 (38.8–79.0)	44.1 (26.3–59.9)	60.3 (51.2–70.8)
Sex (m/f)	6/10	10/4	13/11	15/10	6/9	9/9
Disease duration (years)	9.4^a^ (3.5–16.2)	11.0 (2.1–23.3)	14.5^b^ (2.2–26.6)	11.3^c^ (1.4–25.0)	22.7 (9–37.3)	5.2 (1.3–12.8)
SARA (0–40)	**14.0** (0–21.5)	**13.4** (6.5–22.0)	**12.0** (0–35.0)	**13.3**^d^ (0.5–26.0)	**23.9** (16.5–31.5)	**20.0** (11.5–26.5)
INAS (0–6)	4.19 (0–8)	3.86 (1–7)	4.13 (1–8)	1.79^d^ (0–4)	4.40 (2–7)	4.17 (2–8)
SCAFI (*z*-score)	−**2.54** (−4.34–0.85)	−**2.37** (−4.37 – −1.24)	−**2.28** (−5.40 – −0.08)	−**2.69**^d^ (−5.21 – −0.39)	−**4.2** (−5.1 – −3.2)	−**3.79** (−5.38 – −1.86)
Trinucleotide	CAG	CAG	CAG	CAG	GAA	
Repeats (short allel)	30.38 (28–33)	22.00 (21–23)	21.58 (14–28)	12.36 (10–13)	414.67 (8–1000)	
Repeats (long allel)	46.31 (40–55)	37.50 (35–41)	68.42 (60–76)	22.40 (21–27)	647.73 (200–1000)	
**Control subjects**						
*n*	16	14	24	25	15	18
Age (years)	46.34 (27.0–67.9)	52.2 (34.3–67.8)	53.2 (27.0–76.6)	60.1 (36.2–78.4)	43.2 (24.3–63.6)	61.0 (51.3–71.2)
Sex (m/f)	6/10	10/4	13/11	15/10	6/9	9/9
SARA (0-40)	**0** (0–0)	**0.07** (0–1)	**0.15** (0–2)	**0.16** (0–1)	**0.07** (0–1)	**0.17** (0–2)
SCAFI (z-score)	−**0.152** (−1.11–0.85)	−**0.10** (−1.24–0.98)	−**0.1** (−1.464–0.98)	−**0.33** (−1.98–0.98)	**0.24** (−1.23–1.16)	−**0.34** (−1.464–0.98)

CAG, cytosine–adenine–guanine; GAA, guanine–adenine–adenine.

Cell entries represent mean values across the cohort. Minimum and maximum values across the cohort are specified in parenthesess. SARA, Scale for the Assessment and Rating of Ataxia^16^; INAS, Inventory of non-ataxia symptoms^17^; SCAFI, SpinoCerebellar Ataxia Functional Index.^18^

^a^ Statistics for disease duration was only calculated for 15 of the 16 subjects because one patient was presymptomatic.

^b^ Statistics for disease duration was only calculated for 22 of the 24 subjects because two patients were presymptomatic.

^c^ Statistics for disease duration was only calculated for 23 of the 25 patients because one patient was not aware of the exact start of the disease and the other was presymptomatic.

^d^ One patient was excluded from the descriptive statistics of the clinical ataxia scores because of a confounding comorbidity. This patient and its matched control were excluded from all statistical analyses that considered clinical ataxia scores.

Statistical significance (*P* <  0.001) between a specific ataxia type and the corresponding matched controls assessed using two-sampled *t*-test is indicated by bold font. There were no statistical significances of age between the patient and controls groups.

### Voxel-based susceptibility analysis and VBM

**Figure 1:**
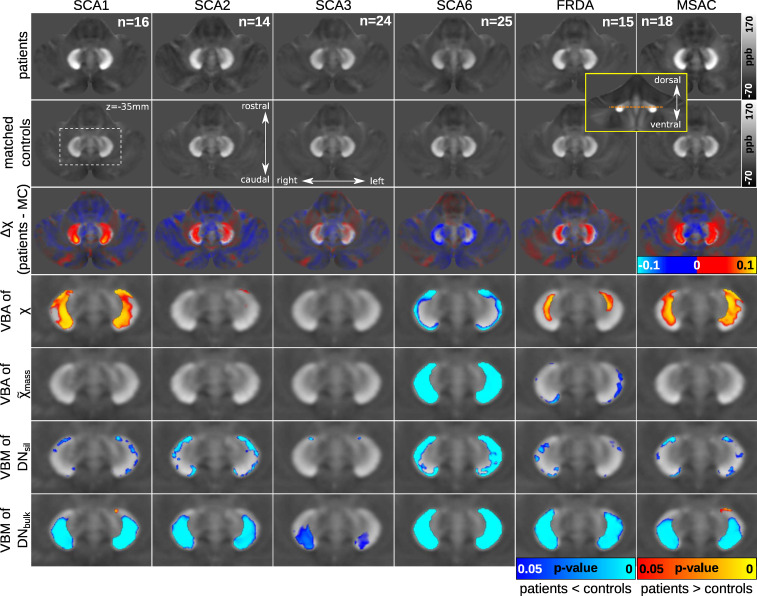
**Mean susceptibility maps and voxel-based statistical analyses for the different ataxia subgroups**. Axial sections cutting the dentate nucleus at the border between the ventral and dorsal dentate nucleus are shown (SUIT space, *z* = −35 mm). The dashed orange line in the inlet surrounded by the yellow frame indicates the location of the axial sections on a coronal slice of a mean susceptibility map computed across all patients and controls included in the study. Rows 1–3: Mean susceptibility maps in the ataxia subgroups (Row 1), the corresponding control groups (Row 2) and the differences between patient and matched control subgroups [Row 3, Δχ(patients—MC)]. Rows 4 and 5: Voxel-based statistical comparisons of susceptibility values (Row 4; VBA of χ), as well as apparent susceptibility mass (Row 5; VBA of ${\tilde{\chi }}_{mass}$) between each subgroup of patients and controls (*P* < 0.05). Rows 6 and 7: Voxel-based statistical comparisons of dentate volumes (VBM) between subgroups of patients and controls (*P* < 0.05). Significant differences in DN_sil_ are shown in Row 6 (VBM of DN_sil_) and significant differences in DN_bulk_ in Row 7 (VBM of DN_bulk_). The statistical maps are superimposed on the mean susceptibility maps of the corresponding control group. The red–yellow colour code represents increases in patients compared with controls and the blue-cyan colour code highlights decreases in patients compared with controls. Left/right, rostral/caudal and dorsal/ventral are the conventions used to describe the localization on the *x*-axis, *y*-axis and *z*-axis in SUIT space, respectively.^39^ The white dashed rectangle (Row 2, Column 1) indicates the location of the sections shown in Rows 4–7. VBA, voxel-based analysis; VBM, voxel-based morphometry. *n*, the number of subjects per group, which was identical for patients and controls within each group. ppb, parts-per-billion; MC, matched controls; DN_sil_, volume-of-interest reflecting the silhouette of the dentate nucleus; DN_bulk_, volume-of-interest reflecting the bulk of iron-rich region of the dentate nucleus.

Voxel-based group analyses between each of the individual ataxia groups and their corresponding controls are summarized in Fig. 1 showing an axial section of the dentate nucleus. The mean susceptibility maps for the patient groups and the corresponding control groups are presented in the first two rows. The absolute difference between these mean susceptibility maps is shown in Row 3. Voxel-wise statistical comparisons between patients and matched controls for susceptibility (*χ*) and apparent susceptibility mass (${\tilde{\chi }}_{mass}$) are displayed in Rows 4 and 5, respectively. As outlined previously, statistical analysis was restricted to the dentate nuclei. Further axial slices of the dentate illustrating the results of the group analyses are presented in Supplementary Figs 3–5.

The most marked differences in susceptibility were found in SCA1, MSA-C and SCA6 patients compared with their matched controls. The pattern of change was very different. While susceptibility was significantly higher in SCA1 and MSA-C patients compared with controls (Row 4, indicated in red to yellow colour scheme, Fig. 1 and Supplementary Figs 3–5), it was significantly lower in SCA6 patients compared with controls (Row 4, indicated in blue to cyan colour scheme, Fig. 1 and Supplementary Figs 3–5). In SCA1 and MSA-C patients, the increased susceptibility was most prominent in the central parts of the middle ventral and lower dorsal dentate nucleus (corresponding to DN_bulk_). Interestingly, this dentate region did not coincide with altered apparent susceptibility masses. The lower susceptibility in SCA6 patients was observed at the surface of the dentate nucleus and was associated with a lower apparent susceptibility mass over the whole dentate (corresponding to DN_bulk_). FRDA patients showed moderately higher magnetic susceptibilities that reached statistical significance in the central parts of the ventro-rostral dentate nuclei (corresponding to DN_bulk_). However, these areas did not coincide with a higher apparent susceptibility mass in the patients. Instead, FRDA patients were found to have small focal areas with lower apparent susceptibility mass on the outer surface of the dentate (corresponding to DN_sil_). Although SCA2 patients also showed numerically higher susceptibility values in the middle ventral and lower dorsal dentate nucleus (Row 3, Fig. 1 and Supplementary Fig. 4), this difference was significant only in a small part of the surface of the left ventro-rostral dentate nucleus (corresponding to DN_sil_, Fig. 1). Furthermore, the apparent susceptibility mass was not altered in SCA2 patients. In SCA3 patients, the difference between patients and controls was very small (Row 3, Fig. 1 and Supplementary Figs 3–5) and did not reach statistical significance for susceptibility (Row 4, Fig. 1 and Supplementary Figs 3–5) and apparent susceptibility mass (Row 5, Fig. 1 and SupplementaryFigs 3–5).

To assess the degree of atrophy of the cerebellar nuclei, volumes of DN_sil_, as a proxy for the volume of the thin wall of the dentate nucleus, and DN_bulk_, comprising the WM within the sac formed by the dentate nucleus, were compared. When DN_sil_ was considered, the most striking change was seen in SCA6 patients, who showed a significant reduction in volume (i.e. decreased extent of regions of increased susceptibility relative to their surroundings; Row 6, Fig. 1 and Supplementary Figs 3–5). This was also the case when DN_bulk_ was considered in SCA6 patients (Row 7, Fig. 1 and Supplementary Figs 3–5). In SCA1, SCA2, FRDA and MSA-C patients, there was a significant reduction in DN_sil_ but much smaller compared with SCA6 patients (Row 6, Fig. 1 and Supplementary Figs 3–5). In contrast, only a very small reduction was observed in DN_sil_ in SCA3 patients (Row 6, Fig. 1 and Supplementary Fig. 4). Considering DN_bulk_, a significant reduction in dentate volume was present in SCA1, SCA2, FRDA and MSA-C patients, and to a lesser degree in SCA3 patients (Row 7, Fig. 1 and Supplementary Figs 3–5). The small regions indicating higher volumes of DN_bulk_ in patients observed at the WM boundary of the left ventro-rostral dentate in SCA1 and MSA-C patients (Row 7, Fig. 1) are most likely caused by methodological constraints, by less accurate spatial alignment at the boundary of the dentate, and should be treated with caution.

**Figure 2:**
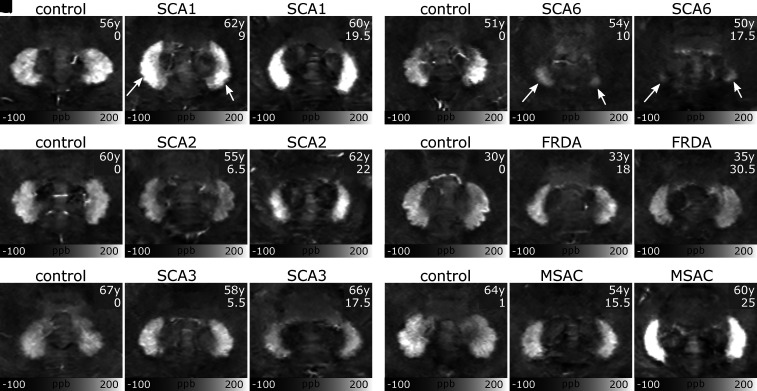
**Examples of susceptibility maps of the dentate nuclei in individual patients and controls**. Patients in each of the ataxia subgroups are set against sex-matched controls of similar age. Healthy controls (**A, D, G, J, M, P**), patients with lower SARA score (**B, E, H, K, N, Q**) and patients with higher SARA score (**C, F, I, L, O, R**) are shown. In controls and most ataxias, the dentate nuclei are clearly discernible due to their high susceptibility. Visual demarcation of dentate nuclei is reduced in SCA6 (arrows). The arrows in **B** indicate regions of higher susceptibility in the dentate. Age (years, y) and SARA score are depicted in the upper right corner in the individual subfigures. Images are presented as average intensity projections over three slices to cover tissue variations typically visible in images with slice thicknesses of 1.5 mm. ppb, parts-per-billion.

Findings are further illustrated exemplarily in Fig. 2, which shows quantitative susceptibility maps of the dentate nuclei in characteristic individual patients together with matched controls. Although the individual-level description is based on visual inspection, the figure provides a representative view of the individual data. For each ataxia type, two patients are shown, one with mild ataxia and another with more severe ataxia as specified by the SARA score (indicated in the upper right corners of the images). Compared with the matched control (Fig. 2A), susceptibility was higher in the SCA1 patients (Fig. 2B and C) and appeared to increase with disease severity. While the clinically less affected MSA-C patient (Fig. 2Q) showed comparable susceptibility to the control (Fig. 2P), the more severely affected patient (Fig. 2R) showed substantially higher susceptibility. The SCA6 patients (Fig. 2K and L) showed reduced volumes and lower susceptibilities than the corresponding control (Fig. 2J). Based on visual inspection, FRDA patients (Fig. 2N and O) showed smaller nuclei compared with the control (Fig. 2M), but little difference in the susceptibility of the dentate, indicating that group-level differences were small. The SCA2 patients (Fig. 2E and F) showed smaller volume than the corresponding control (Fig. 2D), and the susceptibility was higher in the more severely affected patient (Fig. 2F). There was little difference comparing size and susceptibility values between the SCA3 patients (Fig. 2H and I) and the control (Fig. 2G). Dentate nuclei appeared to be smaller in the more severely affected SCA3 patient (Fig. 2I).

**Figure 3:**
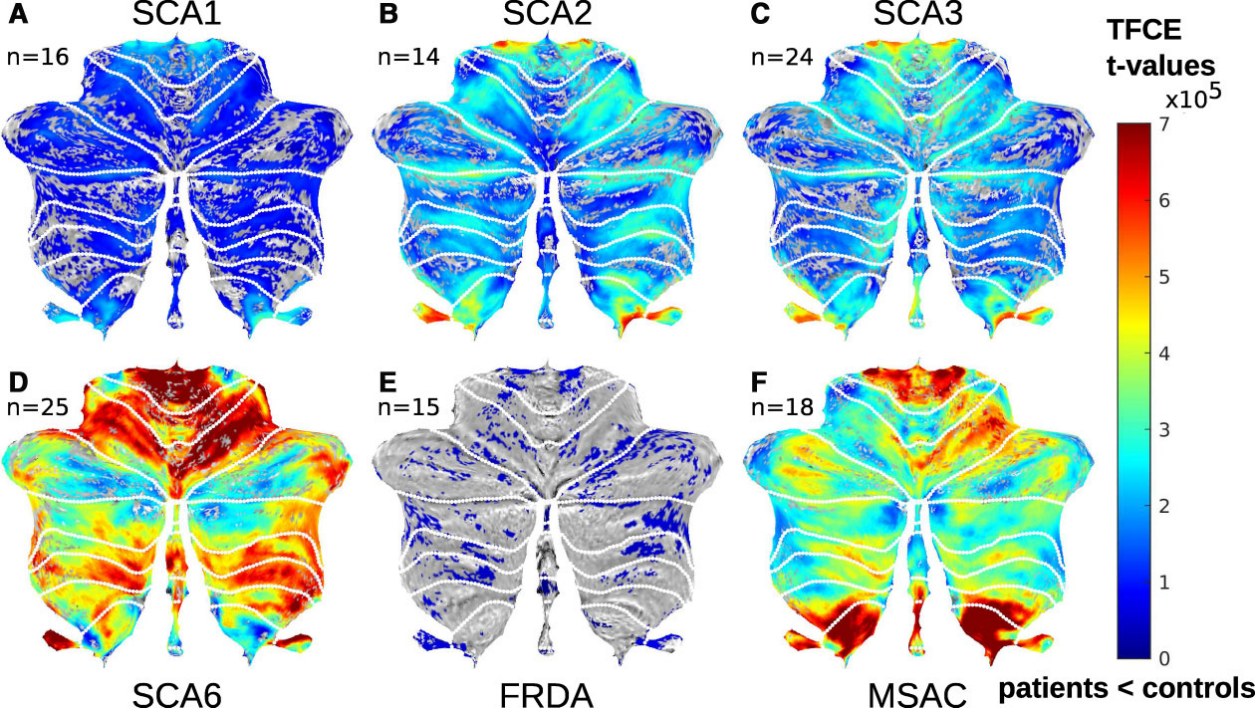
**Voxel-based morphometry of cerebellar grey matter in different ataxia subgroups**. Statistical maps (*t*-statistic) indicating volume reductions in ataxia subgroups compared to the corresponding controls are superimposed on flatmaps of the cerebellum (shown in grey). *T*-values are only shown if TFCE and family-wise error correction yielded *P*-values below 0.05. *n* indicates the number of subjects per group included in the statistical analysis.

The results of the VBM considering cerebellar GM volume are shown in Fig. 3, and superimposed on a flatmap of the cerebellar cortex. Cerebellar GM volume was significantly lower in all ataxia groups compared with matched controls. Consistent with the literature,^23^ FRDA patients exhibited the least cerebellar GM volume reduction, whereas SCA6 and MSA-C patients revealed the greatest reduction. Furthermore, the loss of cerebellar GM volume was more pronounced in SCA2 and SCA3 patients compared with SCA1 patients, and GM loss was less in SCA3 patients compared with SCA2 patients.

### VOI-based analysis

**Figure 4:**
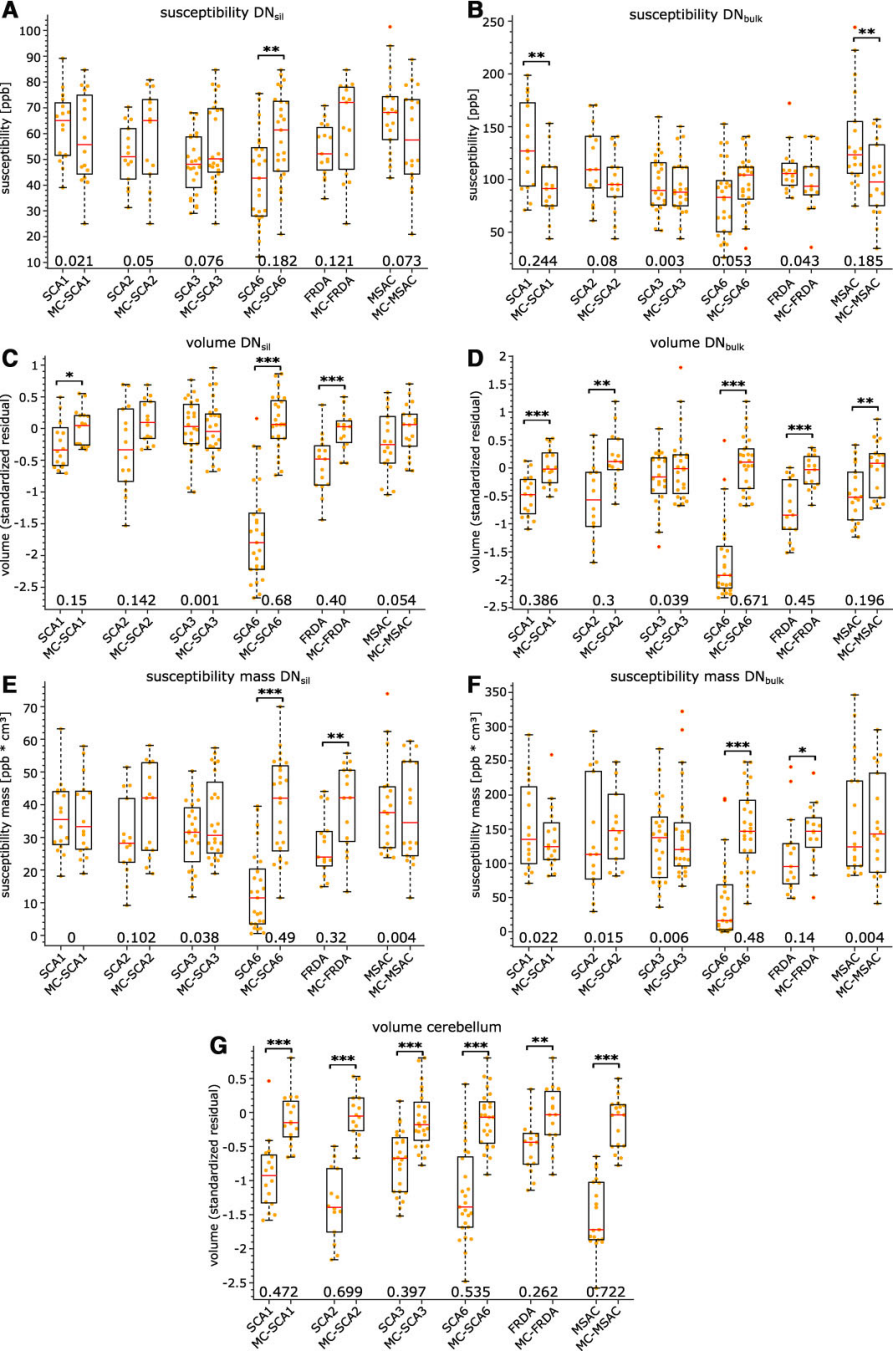
**VOI-based group comparisons for susceptibility, susceptibility mass and volume measures in DN_sil_ and DN_bulk_ as well as the volume of the cerebellum**. Boxplots are shown for the different ataxia subgroups and the matched control groups. Average susceptibilities in DN_sil_ and DN_bulk_ are shown in **A** and **B**, respectively. Standardized residuals of TIV corrected volumes of DN_sil_, DN_bulk_ and cerebellum are presented in **C**, **D** and **G**, respectively (absolute volumes are presented in Table 2). Susceptibility mass measurements of DN_sil_ and DN_bulk_ are depicted in **E** and **F**, respectively. The orange dots indicate individual measurements. Statistical significance between patients and controls is indicated by asterisks (*P < 0.05; **P < 0.01, ***P < 0.001; ANCOVA with a specific patient and control group as group variable and age as covariate). Effect sizes (partial eta squared, *η*^2^) are shown at the bottom. MC, matched controls.

The results of the VOI-based group analysis comparing the ataxia subgroups and their corresponding controls for dentate susceptibility, dentate susceptibility mass, dentate volume and cerebellar volume are summarized in Fig. 4A–G and Table 2.

**Table 2:** Absolute volumes, mean susceptibilities and susceptibility mass values as revealed by VOI-based analysis for each type of ataxia and matched control groups

Group	#Subjects	Volume	Volume	Volume	Volume	Susceptibility	Susceptibility	Susceptibility mass	Susceptibility mass
		Cerebellum	Total in­tra­cranium	DN_sil_	DN_bulk_	DN_sil_	DN_bulk_	DN_sil_	DN_bulk_
		(cm³)	(cm³)	(mm³)	(mm³)	(ppb)	(ppb)	(ppb cm³)	(ppb cm^3^)
SCA1	16	**105.1 **±** 20^+^**	1551.8 ± 137	**568.3 **± 84^*^	**1151.4 **±** 253^#^**	62.7 ± 13	**132.1 **±** 42^+^**	36.1 ± 11	154.1 ± 66
MC-SCA1	16	**126.8 **±** 15^+^**	1545.8 ± 135	**625.6 **± 64^*^	**1477.6 **±** 192^#^**	58.1 ± 1^*^	**93.0 **±** 28^+^**	36.4 ± 12	136.7 ± 46
SCA2	14	**95.5 **±** 18^#^**	1580.7 ± 150	560.6 ± 151	**1119.8 **±** 447^+^**	51.8 ± 12	115.5 ± 36	29.9 ± 12	137.8 ± 84
MC-SCA2	14	**131.0 **±** 14^#^**	1585.9 ± 144	652.6 ± 84	**1600.7 **±** 346^+^**	58.7 ± 19	97.3 ± 29	38.4 ± 14	155.2 ± 58
SCA3	24	**109.5 **±** 15^#^**	1536.6 ± 143	627.3 ± 96	1338 ± 315	48.4 ± 12	95.9 ± 27	31.1 ± 10	133.2 ± 59
MC-SCA3	24	**128.9 **±** 18^#^**	1578.4 ± 159	626.8 ± 92	1499.0 ± 386	56.4 ± 16	93.3 ± 27	35.5 ± 13	142.9 ± 68
SCA6	25	**98.9 **±** 22^#^**	1579.8 ± 162	**265.7 **±** 178^#^**	**436.1 **±** 496^#^**	**42.7 **±** 18^+^**	81.9 ± 34	**13.6 **±** 12^#^**	**45.7 **±** 57^#^**
MC-SCA6	25	**133.0 **±** 16^#^**	1636.3 ± 156	**655.4 **±** 98^#^**	**1552.7 **±** 320^#^**	**59.0 **±** 17^+^**	96.4 ± 26	**39.6 **±** 15^#^**	**152.0 **±** 57^#^**
FRDA	15	**116.9 **±** 13^+^**	1557.6 ± 141	**502.5 **±** 96^#^**	**989.8 **±** 320^#^**	53.7 ± 10	109.1 ± 23	**27.5 **±** 12^+^**	**112.7 **± 58^*^
MC-FRDA	15	**134.1 **±** 16^+^**	1633.8 ± 159	**628.2 **±** 74^#^**	**1465.4 **±** 228^#^**	62.9 ± 18	99.1 ± 29	**39.8 **±** 13^+^**	**144.4 **± 44^*^
MSA-C	18	**88.7 **±** 13^#^**	1538.2 ± 142	571.2 ± 107	**1155.1 **±** 312^+^**	67.8 ± 16	**137.4 **±** 46^+^**	39.3 ± 14	164.4 ± 86
MC-MSA-C	18	**127.9 **±** 15^#^**	1579.9 ± 170	616.2 ± 88	**1450.2 **±** 302^+^**	59.0 ± 19	**100.5 **±** 36^+^**	37.6 ± 15	155.4 ± 76

Values are presented as mean ± standard deviation. The prefix ‘MC’ indicates the corresponding matched control group. Significant differences between a specific ataxia type and the corresponding matched controls are indicated in bold (*P *<* 0.05*,* ^+^*P* *< *0.01*,* ^#^*P* *<* 0.001; ANCOVA with each patients’ and matched controls’ group as group variable and age as covariate). Note that the main statistical analysis is based on volume data normalized for head size as shown in Fig. 4C, D and G. DN_sil_, volume-of-interest that reflects the silhouette of the dentate nucleus; DN_bulk_, volume-of-interest that reflects the bulk of iron-rich region of the dentate nucleus.

VOI-based analysis of susceptibility values reflected the most marked findings of the finer-grained VBA presented previously: susceptibility values were significantly higher in SCA1 and MSA-C patients than in the corresponding controls when DN_bulk_ was considered (Fig. 4B), whereas susceptibility values were significantly lower in SCA6 patients compared with controls when DN_sil_ was considered (Fig. 4A).

Volumes of the dentate nuclei were significantly lower in SCA1, SCA6 and FRDA patients when DN_sil_ was considered (Fig. 4C). When DN_bulk_ was considered, dentate volumes were smaller in SCA1, SCA2, SCA6, FRDA and MSA-C patients compared with matched controls but not in SCA3 patients (Fig. 4D).

The susceptibility masses were significantly lower in SCA6 and FRDA patients considering DN_sil_ (Fig. 4E) and DN_bulk_ (Fig. 4F). Interestingly, the susceptibility masses in SCA1 and MSA-C patients were not statistically different for DN_bulk_ with respect to their corresponding controls (Fig. 4F).

Cerebellar atrophy was present in all ataxia patients (Fig. 4G); however, it was least pronounced in patients with FRDA and SCA3. As mentioned previously, this is in good accordance with the literature.^23^ Although cerebellar volume is often preserved in FRDA patients, volume reduction has been described.^40–42^ As expected, there were no differences in TIVs between patients and controls (Table 2).

### Comparison between ataxia types

ANCOVA revealed ataxia subgroup-specific alterations for dentate susceptibilities, dentate volumes and cerebellar volumes, when including age [dentate volume (DN_sil_): F(5, 105) = 24.104, *P* < 4^−16^, *η*^2^ = 0.534; dentate susceptibility (DN_bulk_): *F*(5,105) = 7.667, *P* = 4e^−6^, *η*^2^ = 0.267; cerebellar volume: *F*(5,105) = 5.812, *P* < 9e^−5^, *η*^2^ = 0.217] and disease severity assessed with SARA [dentate volume (DN_sil_): *F*(5,104) = 29.898, *P* < 1e^−18^, *η*^2^ = 0.590; dentate susceptibility (DN_bulk_): *F*(5,104) = 6.472, *P* = 3e^−5^, *η*^2^ = 0.237 and cerebellar volume: *F*(5,104) = 21.68, *P* < 8e^−15^, *η*^2^ = 0.510] as covariates. *Post hoc* analysis revealed that SCA1 and MSA-C patients had higher dentate susceptibilities compared with SCA3 and SCA6 patients. Furthermore, dentate volumes were significantly lower in SCA6 patients than in all other ataxia subgroups. Cerebellar volumes in FRDA were significantly larger than the other disease types when controlling for SARA. Table 3 summarizes the corresponding *P*-values of the *post hoc* tests and the relationship between each of the ataxia types (i.e. which of the diseases has a higher value than that of the others). Similar findings were observed when looking at the volume of DN_bulk_ and the susceptibility in DN_sil_ (see Supplementary Table 1).

**Table 3:** Comparisons between each of the ataxia types considering dentate susceptibility (DN_bulk_), dentate volume (DN_sil_) and cerebellar volume revealed by ANCOVA

		SCA1	SCA2	SCA3	SCA6	FRDA	MSA-C
Susceptibility DN_bulk_	SCA1	+++	0.93	**0.019** ^a^	**2.2^e^** ^−**4**^ ^a^	0.75	1.0
	SCA2	0.97	+++	0.78	**0.043** ^a^	1.0	0.90
	SCA3	0.04^b^	0.83	+++	0.81	0.94	**0.011** ^b^
	SCA6	**4.9e** ^−**4**^ ^b^	0.088	0.94	+++	0.143	**2.0e** ^−**4**^ ^b^
	FRDA	**0.64**	1.0	1.0	0.66	+++	0.77
	MSA-C	1.0	0.88	**0.02** ^a^	**2.0e** ^−**4**^ ^a^	0.28	+++
Volume DN_sil_	SCA1	+++	1.0	0.82	**5e** ^−**9**^ ^a^	0.86	1
	SCA2	1.0	+++	0.72	**1.3e** ^−**8**^ ^a^	0.96	1.0
	SCA3	0.96	0.82	+++	**1.7e** ^−**15**^ ^a^	**0.026**	0.98
	SCA6	**8.3e** ^−**12**^ ^b^	**2.6e** ^−**10**^ ^b^	**1.0e** ^−**16**^ ^b^	+++	**2.7e** ^−**5**^ ^b^	**1.2e** ^−**11**^ ^b^
	FRDA	1.0	1.0	0.86	**1.0e** ^−**8**^ ^a^	+++	0.65
	MSA-C	1.0	0.99	1.0	**1.7e** ^−**13**^ ^a^	0.97	+++
Volume cerebellum	SCA1	+++	0.63	0.611	1.0	0.51	0.72
	SCA2	**0.041** ^b^	+++	**0.003** ^b^	0.66	**0.004** ^b^	1
	SCA3	1.0	**0.0015** ^a^	+++	0.31	1	0.15
	SCA6	**0.043** ^b^	1.0	**7.6e** ^−**4**^ ^b^	+++	0.39	0.63
	FRDA	**2e** ^−**6**^ ^a^	**7.9e** ^−**12**^ ^a^	**1.2e** ^−**5**^ ^a^	**7.4e** ^−**13**^ ^a^	+++	**0.007** ^a^
	MSA-C	0.68	0.96	**0.14** ^b^	0.99	**1.8e** ^−**11**^ ^b^	+++

*P*-values of the *post hoc* Sidak tests (*α* = 0.05) are shown. The upper triangular matrix shows the *P*-values of the linear relationship considering age as a covariate, whereas the lower triangular matrix depicts the *P*-values of the linear relationship considering SARA as a covariate.

Bold font highlights a *P*-value of <0.05.

^a^ Higher values of volume or susceptibility for the ataxia type specified in the row versus the ataxia type in the column.

^b^ Lower values of volume or susceptibility for the disease type specified in the row versus the disease type in the column. DN_sil_, volume-of-interest that reflects the silhouette of the dentate nucleus. DN_bulk_, volume-of-interest that reflects the bulk of the iron-rich region of the dentate nucleus. +++, delimiter to separate the lower triangular matrix from the upper triangular matrix.

### Correlation with ataxia scores

**Figure 5:**
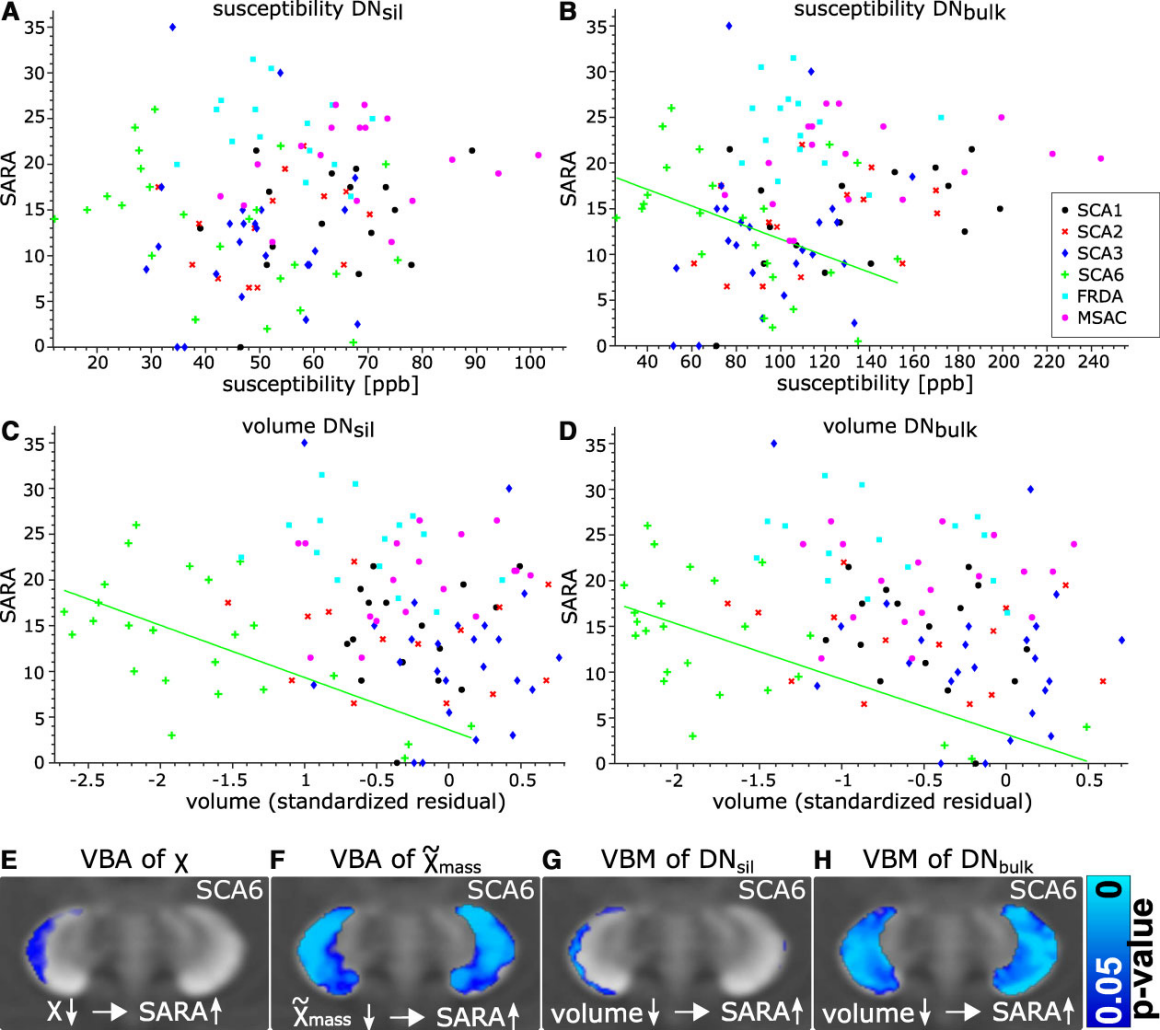
**Correlation analysis.** SARA scores are plotted as function of susceptibility (**A**, **B**) and volume (**C**, **D**) measures in DN_sil_ and DN_bulk_. Note that significant correlations (corrected for age) were observed only in SCA6 patients (**B, C, D**; *P* < 0.05). This is further illustrated in **E, F, G, H**, which show significant voxel-wise correlations in SCA6 patients (*P* < 0.05). (**E**) Voxel-based correlations between susceptibility and SARA (corrected for age) and (**F**) between apparent susceptibility mass and SARA (corrected for age). (**G, H**) Voxel-based morphometry between DN_sil_ and SARA as well as DN_bulk_ and SARA (both corrected for age and TIV), respectively. VBA, voxel-based analysis; VBM, voxel-based morphometry.

Dentate susceptibility and dentate volume correlated with SARA scores in SCA6 patients at both the VOI-based and voxel-based levels. At the voxel-based level, the susceptibilities of the surface of the right dentate (Fig. 5E), the susceptibility mass (Fig. 5F) and the volumes of DN_sil_ (Fig. 5G) and DN_bulk_ (Fig. 5H) correlated inversely with SARA scores. Likewise, VOI-based analysis revealed that the dentate volumes (Spearman’s correlation; DN_sil_: *r* = −0.5, *P* = 0.016; DN_bulk_: *r* =  −0.51, *P* = 0.012) and the susceptibilities in DN_bulk_ (Spearman’s correlation; *r* = −0.42, *P* = 0.046) were inversely correlated with SARA when age was included as a covariate. The susceptibilities in DN_sil_ showed a trend to moderate correlation but did not reach statistical significance (*r* = −0.40, *P* =  0.056). None of the correlations were significant in any of the other ataxia types (see also Fig. 5A–D).

## Discussion

In the present study, volumes of the cerebellar dentate nuclei were determined and magnetic susceptibility was assessed by QSM for common types of hereditary and non-hereditary degenerative ataxias. We found marked elevation of susceptibility in SCA1 and MSA-C, a moderate elevation in FRDA, and a tendency of elevated susceptibility in SCA2, while susceptibility was found to be lower in SCA6 and to be unaltered in SCA3. Atrophy of the dentate nuclei was observed to various degrees in all ataxias, but was most marked in SCA6. Susceptibility mass was lower in SCA6 and FRDA and unchanged in the other ataxia types studied.

The mean susceptibility and susceptibility mass measures were evaluated as proxies for iron concentration and total iron content, respectively. As outlined in more detail below, iron content is known to be high in glial cells but not in neurons of the dentate nuclei. Thus, one interpretation of the current findings is that changes in iron-rich glial cells may contribute to the pathology of a subset of ataxias. Changes in iron concentration, however, are not necessarily indicative of iron accumulation (or loss) but may also result from a reduction (or increase) of cells with low iron content.^26^ Furthermore, although susceptibility is a very sensitive measure of brain iron concentration,^14,43^ it is not specific. Accumulation of other paramagnetic materials such as copper (II) or manganese compounds^44^ and myelin loss also cause high susceptibility,^45^ whereas calcifications cause low susceptibility.^46^ The interpretations of our findings can only be indirect and need to be confirmed in comparative QSM and histopathological studies.

The most striking observation of the present study was that susceptibility in dentate nuclei was significantly higher in SCA1 and MSA-C patients than in controls. Our findings of higher susceptibility in MSA-C are in good agreement with previous findings in the literature.^47^ At first glance, the cellular pathogenesis of these two diseases has little in common. In SCA1, there is marked neuronal loss in both the cerebellar cortex and nuclei,^48^ whereas in MSA, it is mainly the oligodendrocytes that are affected.^49^ In both diseases, however, pronounced microgliosis has been described.^10,50–53^ We hypothesize, but QSM does not allow us to provide direct evidence that microgliosis contributes, at least in part, to the high susceptibility values in the dentate nuclei in SCA1 and MSA-C. In the healthy cerebellum, the high iron content in the cerebellar nuclei mainly reflects ferritin in oligodendrocytes.^54^ The known age-related increase in iron content in cerebellar nuclei is mainly due to microglia.^54^ In the diseased brain, iron accumulates as well in microglia—with iron accumulation thought to increase the risk for oxidative stress as a possible cause for neuronal death. Additional inflammatory processes may also play a role.^55,56^ Significant glial activation has been described in post-mortem histology of various ataxias.^51^ It has been studied most extensively in SCA1 patients^57,58^ and likely plays an important role in the pathogenesis of SCA1.^10^ In mouse models of SCA1, activation of microglia occurred early in the disease and was present prior to neuronal cell death.^10^ In fact, reduction of microglia early in the disease resulted in an amelioration of motor deficits.^52^ Glial markers have been proposed as early biomarkers of SCA1. Likewise, the present findings suggest that MR-based iron measures in the cerebellar nuclei may allow us to assess activation of microglia in SCA1 *in vivo*. As outlined previously, the loss of myelin could also contribute.

MSA, on the other hand, is considered to be a primary oligodendrogliopathy. Due to the known high iron content of oligodendroglia, which is required for the production and maintenance of myelin,^59^ impaired iron metabolism is thought to be involved in the pathogenesis of MSA-associated neurodegeneration.^7^ In fact, increased iron content and microglial proliferation in the basal ganglia have been described in the Parkinson variant of MSA.^50^ Apoptosis of oligodendroglia has been reported, and iron may shift from oligodendroglia to microglia.^60^ Our findings suggest that a similar pathomechanism may apply to MSA-C with increased iron concentration in cerebellar nuclei. This is in good agreement with a previous post-mortem study in MSA-C patients that reported a diffuse increase of ferritin in the cerebellar nuclei, with neurons being largely preserved.^6^ MSA-C is accompanied by widespread demyelination, which may also contribute to increased susceptibility.^45^

Of note, the higher susceptibility in SCA1 and MSA-C patients was accompanied by mild-to-moderate atrophy of the dentate nuclei. Taking into account the degree of atrophy of the dentate nuclei, the difference in susceptibility between patients and controls disappeared. In other words, compared with controls susceptibility masses were unaltered in SCA1 and MSA-C patients, indicating higher iron concentration but unchanged iron content. Thus, a reduction of cells containing less iron, i.e. neurons and/or astroglia, is likely to contribute to the present findings. The mean susceptibilities and non-normalized volumes of the denate, however, did not correlate (Pearson correlation; SCA1: *r*_DNsil _= 0.42, *p*_DNsil_ = 0.10, *r*_DNbulk_ =  0.2, *p*_DNbulk_ = 0.45; MSA-C: *r*_DNsil_ _ _= 0.36, *p*_DNsil_ = 0.15, *r*_DNbulk_ = 0.42, *p*_DNbulk_ = 0.08). It is therefore unlikely that the increased susceptibility can be explained by a reduction of non-iron-rich cells alone.

In contrast to SCA1, only a trend towards higher susceptibility in dentate nuclei was observed in SCA2 and no obvious susceptibility change was observed in SCA3, while susceptibility masses remained unaltered. The influx of iron-rich microglia may be less pronounced in SCA2 and SCA3 than in SCA1. As described previously, the present findings need to be confirmed in future histological studies.

A moderately higher susceptibility was found in more circumscribed parts of the dentate nuclei in FRDA. However, at the level of the entire dentate (DN_bulk_), susceptibilities were not significantly different from controls (Fig. 4 and Table 2). The latter finding is consistent with previous results from our group^61^ using T_2_- and T_2_^*^-relaxometry but is at variance with data reported by others using relaxometry^62,63^ and QSM.^64^ The present finding of a more localized increase in iron concentration in the dentate nuclei with FRDA, however, agrees well with *post-mortem* data.^65^ Although there is good evidence that iron homeostasis is disturbed in FRDA, there is no clear evidence that iron accumulates in the diseased human brain.^8^ Studies in yeast have shown that frataxin deficiency leads to iron accumulation in the mitochondria.^66^ In FRDA patients, however, clear evidence of iron (i.e. ferritin) accumulation was only found in the heart.^67^ Total iron and ferritin content in dentate nuclei of FRDA patients is not different from controls.^65^ Because the cerebellar nuclei are reduced in size,^61,64,65^ a finding confirmed in the present study, higher susceptibility values may reflect increased iron concentration rather than its accumulation.^68^ Taking the degree of atrophy of the dentate nuclei into account, susceptibility mass was even lower (in particular at the outer dentate surface), indicating lower total iron content in FRDA patients compared with controls.

A loss of oligodendrocytes and a shift of iron from oligodendrocytes to microglia, but also astrocytes, has been described in the dentate nucleus in FRDA.^65^ Thus, microgliosis might also play a role.^69^ In the present study, higher susceptibilities were most prominent in the WM located within the thin walls of the ventro-rostral dentate nucleus, whereas apparent susceptibility mass was not different (Fig. 1). Histological findings indeed show that oligodendrocytes in the hilus are smaller and more densely packed in FRDA patients, likely due to a loss of myelin.^69^ This observation is in line with the assumption of regional differences in iron concentration. Reduced myelin content, however, also leads to higher susceptibility, so demyelinization may also have contributed to the present findings.

Atrophy of the cerebellar nuclei was found to varying degrees in all ataxias included in the present study. It was most pronounced in patients with SCA6, in good accordance with previous findings by Stefanescu *et al*.^23^ For these patients, we observed a significant correlation between non-normalized dentate volumes and measured mean susceptibilities (Pearson correlation; *r*_DNsil_ = 0.75, *p*_DNsil_ < 0.001, *r*_DNbulk_ _ _= 0.6, *p*_DNbulk_ < 0.002). SCA6 typically manifests as a relatively pure cerebellar phenotype compared to SCA1, SCA2 and SCA3 and is thought to be caused primarily from degeneration of Purkinje cells in the cerebellar cortex.^70^ Mouse models with pure Purkinje cell degeneration also show significant atrophy of the cerebellar nuclei.^71,72^ While the loss of neurons, likely due to trans-synaptic degeneration, and reactive astrogliosis has been described in the cerebellar nuclei in SCA6 patients,^73,74^ it is unlikely that this is the main cause of volume reduction. First, neurons make up only a small fraction of the volume of neuronal tissue; e.g. only 8% of the volume of the cerebellar nuclei are neurons in wild-type mice.^71^ Second, as outlined previously, iron is most abundant in oligodendrocytes, and neuronal loss cannot explain a decrease in both susceptibility and susceptibility mass in the dentate nuclei in SCA6. The simplest explanation would be a reduction of oligodendrocytes. In fact, in Lurcher mice, a model of pure Purkinje cell degeneration, it was found that the concomitant atrophy of the nuclei could be explained largely by a loss of ‘myelinated axons and boutons’ (accounting for 59% of the atrophy) and a loss of ‘glial processes, vascular elements, and intercellular space’ (accounting for 30.7%).^71^ The loss of neurons accounted for only 2% of the atrophy. The loss of myelin, however, is expected to increase susceptibility.^45^ Microcalcifications in the dentate may also play a role, resulting in lower susceptibility, but this has never been investigated. Future histological studies are needed to explain the reduced susceptibility of the nuclei in SCA6 patients. Furthermore, Purkinje cell degeneration is not limited to SCA6 but also occurs in other ataxias included in the present study, particularly SCA1 and SCA2. One possible reason for the differences in susceptibility and atrophy of the dentate nuclei could be differences in the degree of concomitant microgliosis.

The degree of atrophy of the dentate nuclei was least pronounced in SCA3 patients. Apart from a very small area ventral-rostrally of DN_sil_ (Fig. 1), significant atrophy was only found on the basis of VBM of DN_bulk_ and only in parts of the WM located within the thin GM ribbon of the dentate nucleus. This is surprising given that neurons in the dentate nuclei are known to undergo severe degeneration.^75^ Marked astrogliosis could be one reason why neuronal atrophy is not revealed by QSM.^75^ Unlike a previous study by our group,^23^ SCA3 patients did not show reduced volume based on tracing of the silhouette of the dentate nucleus (DN_sil_). Reanalysis of the previous data set showed that different strategies had been applied in manual delineation of the nuclei.

This points to one of the limitations of MRI-based volumetry of cerebellar nuclei. Despite attempts at automatic segmentation of the cerebellar nuclei,^76–78^ manual delineation remains the gold standard to quantify atrophy of the dentate nuclei. The iron-rich dentate area shown on MR images extends beyond the extent of the thin highly corrugated wall of the dentate nucleus seen on histology (see Supplementary Fig. 1A and B),^24,25^ making it difficult to clearly identify the dentate *in vivo*. As yet, accurate differentiation of the thin wall of the human dentate nuclei from adjacent WM has only been achieved in MR images *ex vivo* (see Fig. 1 in Sereno *et al*.^25^). We evaluated the reliability of our cerebellar nuclei VOI definitions (DN_sil_, DN_bulk_) in the group of SCA6 patients and their matched controls (*n* = 50) by analysing the volumes and average susceptibilities of each hemisphere provided by these two VOI definitions using intraclass correlation coefficients (ICCs, two-way mixed-effects model)^79^ between two raters (inter-rater) and between repeated demarcations (intra-rater), respectively. Intra-rater reliability assessed via ICCs separately for two independent raters was always above 0.82 (except for the right DN_sil_ of the second rater with an ICC of 0.69) when considering volumes and susceptibilities of the two regions DN_sil_ and DN_bulk_. For the same subjects, the corresponding ICCs between two independent raters were always above 0.76. The analyses throughout this study were performed on the dentate VOI definitions of rater 1 for whom according to Koo and Li^79^ good reliability across SCA6 patients and matched controls was achieved. A detailed investigation of intra-rater and inter-rater reliability will be published elsewhere.

Another limitation of our study, as already highlighted previously, is that the biophysical origin of susceptibility is not unequivocally known. While the measure of susceptibility is highly sensitive to iron concentration, it is also affected by other biophysical origins such as myelin, calcium or copper. Furthermore, increases in susceptibility do not allow us to differentiate between iron accumulation, i.e. an influx of iron, or increased iron concentration due to a reduction of cells containing little iron. Therefore, the interpretations of our findings are only indirect and need to be confirmed in comparative QSM and histopathological studies.

Clinical correlations were significant only in SCA6 patients. This finding is in good agreement with a previous study by our group that showed a negative correlation between dentate volume and ataxia scores in SCA6 patients but not in SCA3 and FRDA patients.^23^ Because the clinical SARA score is not specific to cerebellar symptoms (e.g. sensory ataxia also leads to an elevated SARA score) but also to many other types of motor dysfunction, it exclusively represents cerebellar dysfunction in the case of pure cerebellar disease.^16^ Therefore, we hypothesize that the significant correlations with SARA present only in SCA6 are due to the fact that SCA6 is a purer form of cerebellar degeneration, whereas the other ataxia subtypes have significant extracerebellar pathology that also contributes to the SARA score. However, the cohorts studied are small with heterogeneous samples and may have been underpowered for correlations.

## Conclusions

QSM revealed abnormalities of the dentate nuclei in common types of hereditary and non-hereditary ataxias. The most striking alterations in susceptibilitiy were found in SCA1 and MSA-C and in SCA6. Higher susceptibility and unchanged susceptibility mass in the dentate nuclei in SCA1 and MSA-C suggest a reduction in neurons (increase in iron concentration) and/or an increase in iron-rich glial cells, e.g. microgliosis. Demyelinization may also contribute. The lower susceptibility in SCA6 confirms previous studies and suggests a loss of iron-rich glial cells. The QSM data warrant future studies of iron content and iron-rich cells in the dentate nuclei in cerebellar ataxias to gain a more comprehensive understanding of the pathogenesis of these diseases, as well as to explore additional biomarkers and treatment options.

## Supplementary Material

fcab306_Supplementary_Data

## Abbreviations

ANCOVA =: analysis of covariance
QSM =: quantitative susceptibility mapping
DN_bulk_ =: volume-of-interest reflecting the bulk of the iron-rich region of the dentate nucleus
DN_sil_ =: volume-of-interest reflecting the silhouette of the dentate nucleus
FRDA =: Friedreich’s ataxia
GM =: grey matter
MSA =: multiple system atrophy
MSA-C =: multiple system atrophy, cerebellar type
SCA =: spinocerebellar ataxia
SUIT =: spatially unbiased infra-tentorial template
TIV =: total intracranial volume
VBA =: voxel-based analysis
VBM =: voxel-based morphometry
VOI =: volume-of-interest
WM =: white matter
