# Supplementary material for: Quantitative susceptibility mapping reveals alterations of dentate nuclei in common types of degenerative cerebellar ataxias
Source: Brain Commun. 2022 Jan 13;4(1):fcab306. doi: 10.1093/braincomms/fcab306 (PMC8914888; doi:10.1093/braincomms/fcab306)
Supplement: fcab306_Supplementary_Data [file fcab306_Supplementary_Data.pdf]

Supplementary material

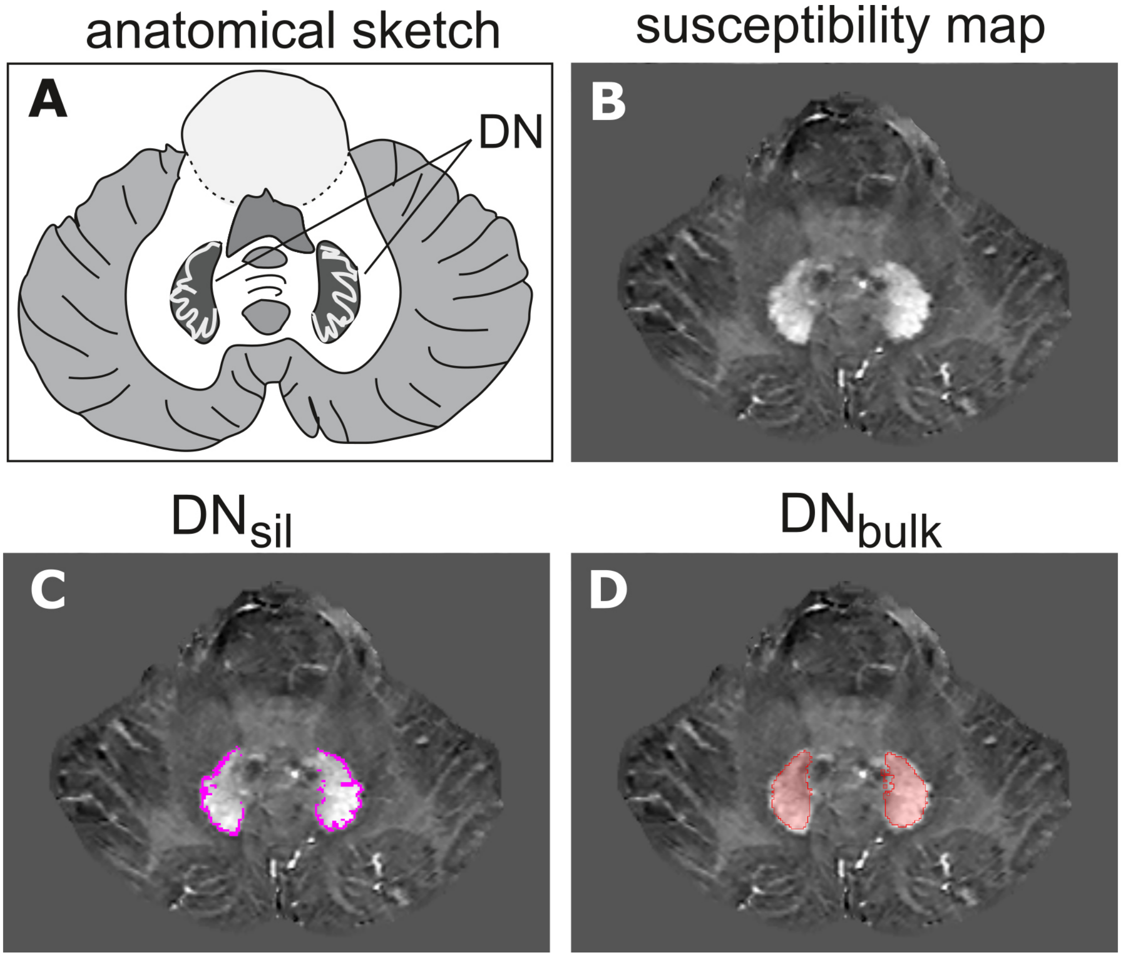

Supplementary Figure 1: Depiction of the two dentate volumes-of-interest, DN$_{sil}$ and DN$_{bulk}$, in axial images of the cerebellum. (A) Sketch of the appearance of the dentate nucleus (DN) on T_2^*-weighted MR images *ex vivo*.[1] (B) Quantitative susceptibility map in a healthy female participant (54 years of age). (C) DN$_{sil}$ indicated in purple. DN$_{sil}$ was manually traced and followed the silhouette of the dentate nucleus. (D) DN$_{bulk}$ indicated in red. DN$_{bulk}$ was automatically calculated based on the convex hull (thin red line) obtained from DN$_{sil}$.

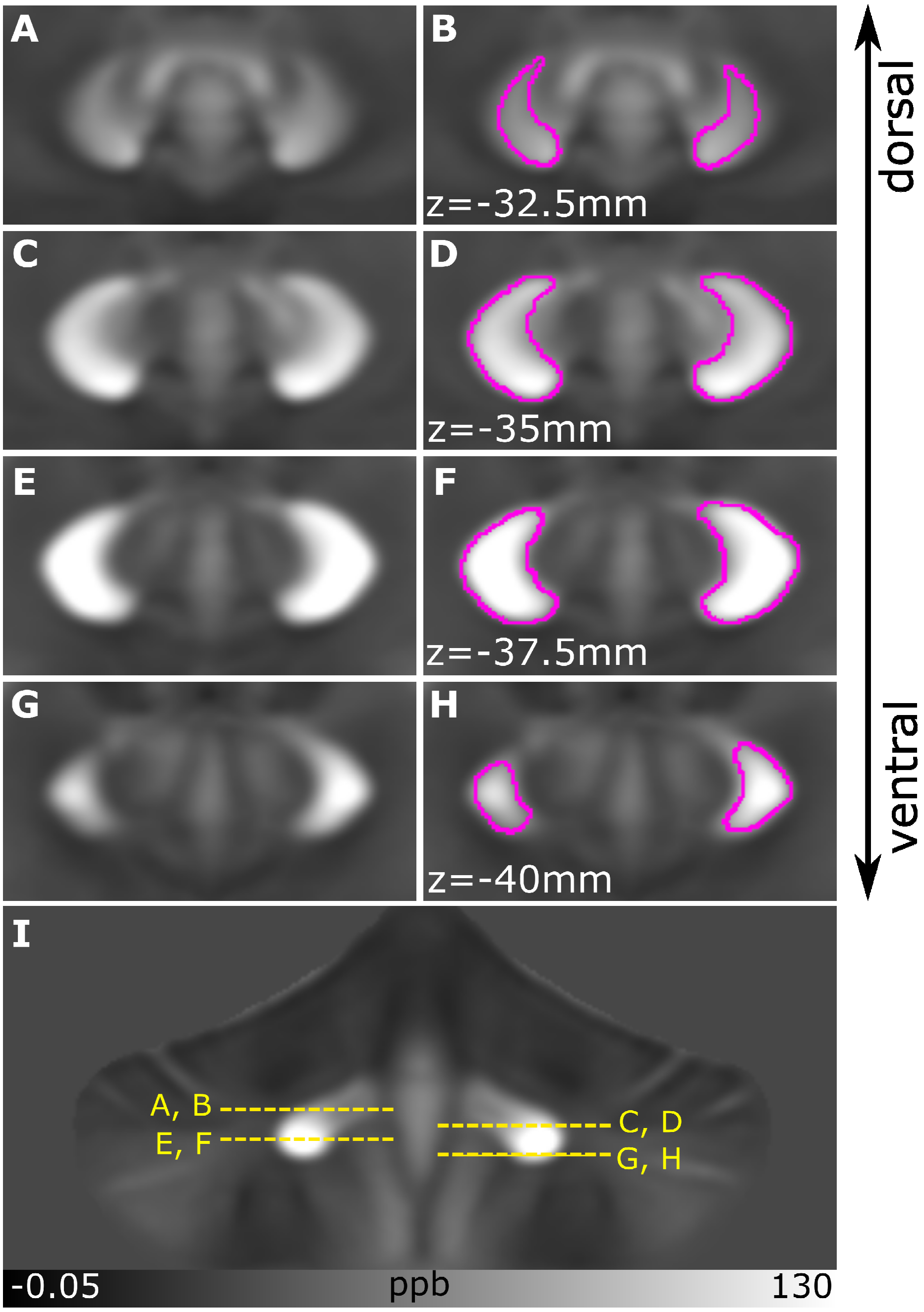

Supplementary Figure 2: Dentate nucleus mask considered for voxel-based statistical analysis. Susceptibility maps averaged across all patients and controls included in the study. Axial views without dentate mask are shown in (A), (C), (E), and (G) and with dentate mask (used for statistical analysis) as overlay are presented in (B), (D), (F), and (H). The location of the axial slice positions in SUIT space is specified at the bottom in (B), (D), (F), and (H) and is indicated by yellow dashed lines with corresponding subfigure labels on the coronal mean susceptibility map in (I). The dentate mask has been provided by the SUIT toolbox and was manually corrected to ensure that the whole dentate is captured without including the surrounding white matter.

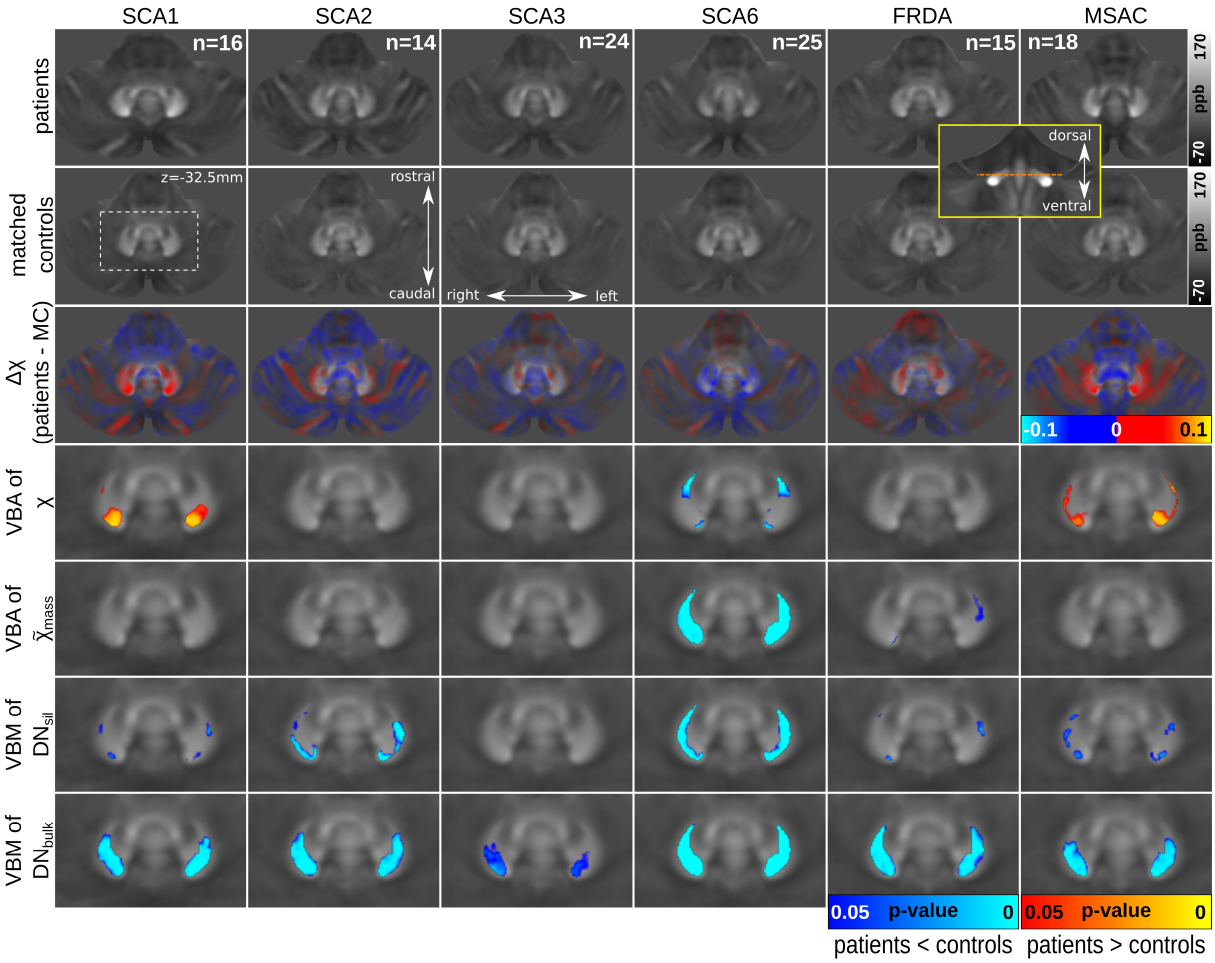

Supplementary Figure 3: Voxel-based analysis of the middle dorsal part of the dentate nuclei. Mean susceptibility maps and voxel-based statistical analyses for the different ataxia subgroups in axial view showing the middle dorsal part of the dentate nuclei (SUIT-space, z = -32.5 mm). The dashed orange line in the inlet surrounded by the yellow frame indicates the location of the axial sections on a coronal slice of a mean susceptibility map computed across all patients and controls included in the study. Rows 1-3: Mean susceptibility maps in the ataxia subgroups (row 1), the corresponding control groups (row 2), and the differences between patient and matched control subgroups (row 3, Δχ(patients – MC)). Rows 4, 5: Voxel-based statistical comparisons of susceptibility values (row 4; VBA of χ) as well as apparent susceptibility mass (row 5; VBA of $\tilde{\chi}_{mass}$) between each subgroup of patients and controls ($p < 0.05$). Rows 6, 7: Voxel-based statistical comparisons of dentate volumes (VBM) between subgroups of patients and controls ($p < 0.05$). Significant differences in DN_{sil} are shown in row 6 (VBM of DN_{sil}), and significant differences in DN_{bulk} in row 7 (VBM of DN_{bulk}). The statistical maps are superimposed on the mean susceptibility maps of the corresponding control group. The red-yellow color code represents increases in patients compared to controls and the blue color code highlights decreases in patients compared to controls. Left/right, rostral/caudal, and dorsal/ventral are the conventions used to describe the localization on the x-axis, y-axis and

z-axis in SUIT space, respectively [2]. The white dashed rectangle (row 2, column 1) indicates the location of the sections shown in rows 4 to 7. VBA – voxel-based analysis. VBM – voxel-based morphometry. n – the number of subjects per group, which was identical for patients and controls within each group. ppb – parts-per-billion. MC – matched controls. DN_{sil} – volume-of-interest reflecting the silhouette of the dentate nucleus. DN_{bulk} – volume-of-interest reflecting the bulk of iron-rich region of the dentate nucleus.

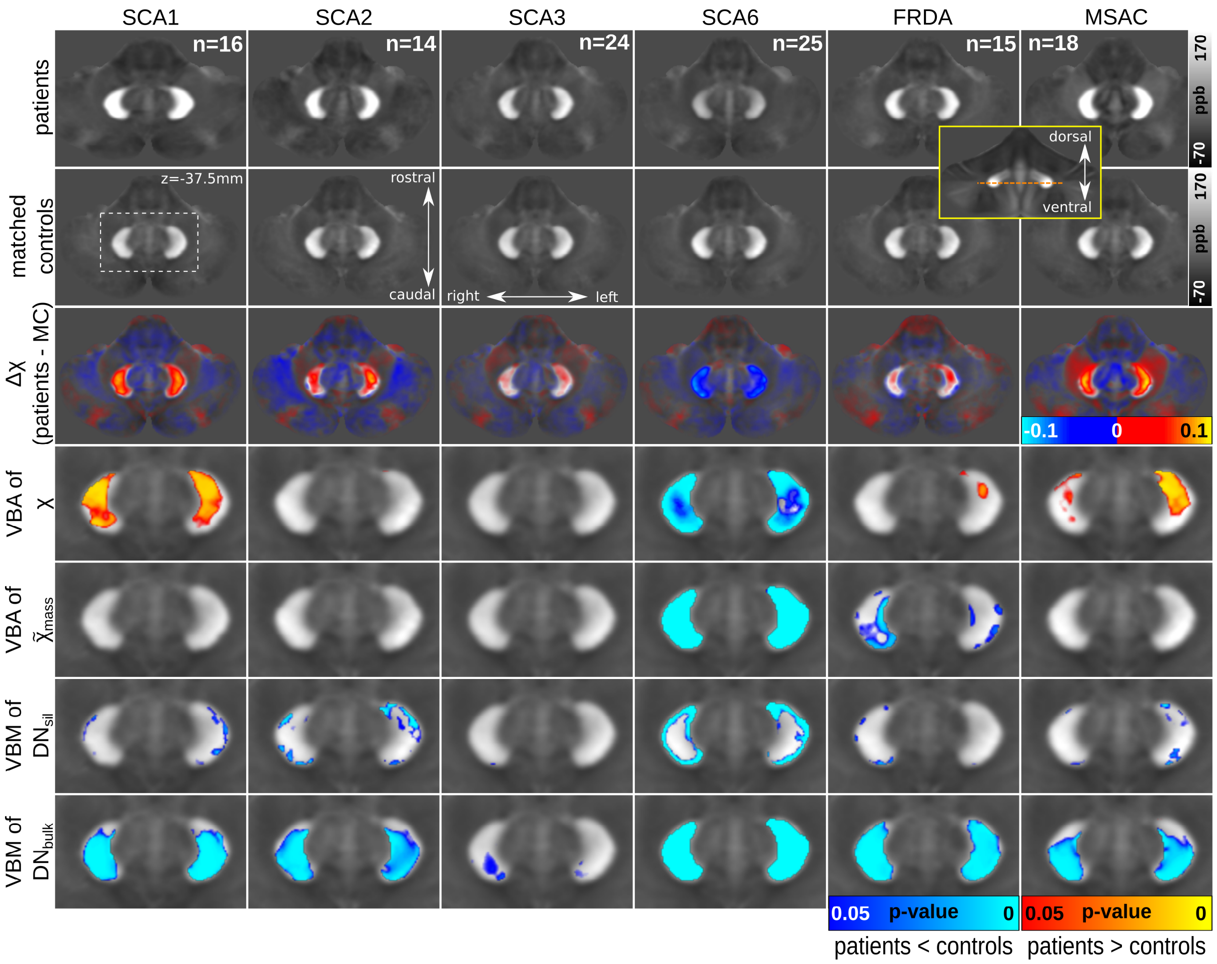

Supplementary Figure 4: Voxel-based analysis of the middle part of the ventral dentate nuclei. Mean susceptibility maps and voxel-based statistical analyses for the different ataxia subgroups showing an axial section of the more middle part of the ventral dentate nuclei in axial view (SUIT-space, z = -37.5 mm). Additional information on the presented subimages and details can be found in the figure caption of Supplementary Figure 3.

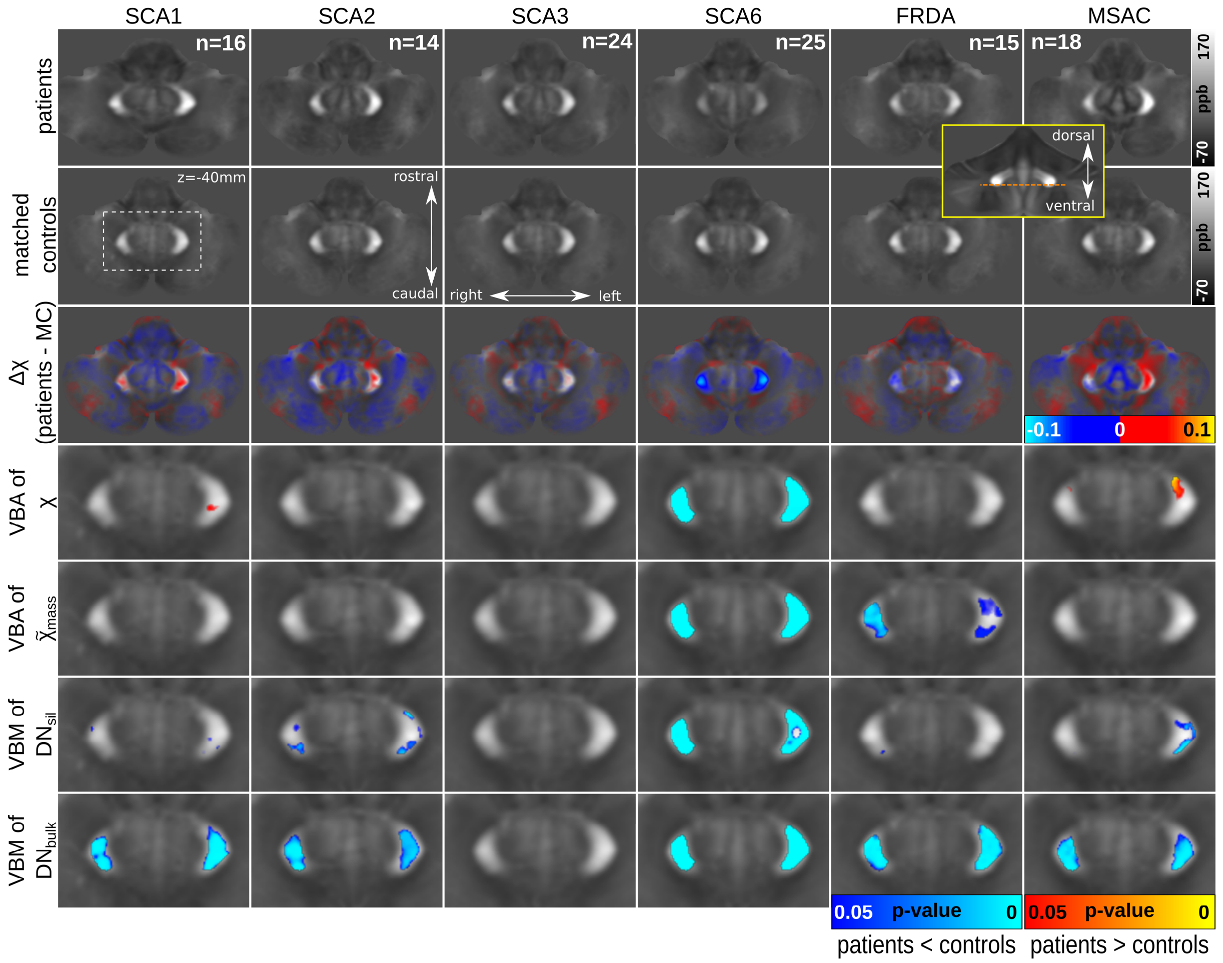

Supplementary Figure 5: Voxel-based analysis of the lower part of the ventral dentate nuclei. Mean susceptibility maps and voxel-based statistical analyses for the different ataxia subgroups in axial view showing the lower ventral aspect of the dentate nuclei (SUIT-space, z = -40 mm). Additional information on the presented subimages and details can be found in the figure caption of Supplementary Figure 3.

Supplementary Table 1: Comparisons between each of the ataxia types considering dentate susceptibility (DN_{sil}) and dentate volume (DN_{bulk}) as revealed by ANCOVA. Differences between ataxia types were detected by ANCOVA considering age as covariate (dentate susceptibility [DN_{sil}]: $F(5, 105)=8.745$, $p < 5e-7$, $\eta^2=0.294$; dentate volume [DN_{bulk}]: $F(5, 105)=16.657$, $p <4e-12$, $\eta^2=0.442$) and SARA-score as covariate (dentate susceptibility [DN_{sil}]: $F(5, 104)=8.631$, $p = 8e-7$, $\eta^2=0.293$; dentate volume [DN_{bulk}]: $F(5, 104)=21.875$, $p < 7e-15$, $\eta^2=0.513$), respectively. p-values of the post-hoc Sidak test ($\alpha=0.05$) are shown. The upper triangular matrix shows the p-values of the linear relationship considering age as a covariate, whereas the lower triangular matrix depicts the p-values of the linear relationship considering SARA as a covariate.

		SCA1	SCA2	SCA3	SCA6	FRDA	MSA-C
susceptibility DN_{sil}	**SCA1**	+++	0.41	**0.03$^{\#}$**	**6.5e$^{-4\#}$**	0.721	0.999
	**SCA2**	0.41	+++	1.0	0.551	1.0	**0.04§**
	**SCA3**	**0.025§**	1.0	+++	0.907	0.983	**6.3e$^{-4\S}$**
	**SCA6**	**2.5e$^{-4\S}$**	0.524	0.923	+++	0.294	**1e$^{-6\S}$**
	**FRDA**	0.957	1.0	0.932	0.166	+++	0.172
	**MSA-C**	0.977	0.2	**3.8e$^{-4\#}$**	**1e$^{-6\#}$**	0.12	+++
volume DN_{bulk}	**SCA1**	+++	1.0	0.761	**3e$^{-6\#}$**	0.964	1.0
	**SCA2**	1.0	+++	0.558	**1e$^{-5\#}$**	0.998	1.0
	**SCA3**	0.93	0.67	+++	**3.8e$^{-12\#}$**	**0.046$^{\#}$**	0.933
	**SCA6**	**9.4e$^{-9\S}$**	**3.4e$^{-7\S}$**	**7.4e$^{-14\S}$**	+++	**1.6e$^{-3\S}$**	**2.9e$^{-8\S}$**
	**FRDA**	1.0	1.0	0.996	**2.2$^{e-7\#}$**	+++	0.875
	**MSA-C**	0.994	0.909	1.0	**6.2e$^{-11\#}$**	0.999	+++

Bold font – highlights a p-value less than 0.05. $^{\#}$ – indicates higher values of volume or susceptibility for the ataxia type specified in the row vs. the ataxia type in the column. § – indicates lower values of volume or susceptibility for the disease type specified in the row vs. the disease type in the column. DN_{sil} – volume-of-interest that reflects the silhouette of the dentate nucleus. DN_{bulk} – volume-of-interest that reflects the bulk of the iron-rich region of the dentate nucleus. +++ – delimiter to separate the lower triangular matrix from the upper triangular matrix.

References

1. Sereno MI, Diedrichsen J, Tachrount M, Testa-Silva G, d'Arceuil H, De Zeeuw C. The human cerebellum has almost 80% of the surface area of the neocortex. *Proc Natl Acad Sci U S A*. Aug 11 2020;117(32):19538-19543. doi:10.1073/pnas.2002896117

2. Dum RP, Strick PL. An unfolded map of the cerebellar dentate nucleus and its projections to the cerebral cortex. *Journal of neurophysiology*. Jan 2003;89(1):634-9. doi:10.1152/jn.00626.2002
